# Jejunal Leiomyosarcoma Diagnosed via Anterograde Enteroscopy: Case Report of a Rare Presentation of Obstructive Small Bowel Tumor

**DOI:** 10.7759/cureus.95080

**Published:** 2025-10-21

**Authors:** Ygor R Fernandes, Robson K Ishida, Vitor R Grossi, Fauze Maluf Filho

**Affiliations:** 1 Gastrointestinal Endoscopy, Hospital das Clínicas da Faculdade de Medicina da Universidade de São Paulo (HCFMUSP), São Paulo, BRA; 2 Digestive Endoscopy, Heart Institute, Hospital das Clínicas da Faculdade de Medicina da Universidade de São Paulo (HCFMUSP), São Paulo, BRA; 3 Pathology, Hospital das Clínicas da Faculdade de Medicina da Universidade de São Paulo (HCFMUSP), São Paulo, BRA; 4 Gastroenterology, Cancer Institute of the State of São Paulo, Hospital das Clínicas da Faculdade de Medicina da Universidade de São Paulo (HCFMUSP), São Paulo, BRA

**Keywords:** double-balloon enteroscopy, endoscopic diagnosis, gastrointestinal sarcoma, jejunal leiomyosarcoma, small bowel neoplasms

## Abstract

Primary leiomyosarcoma (LMS) of the small intestine is an uncommon and aggressive malignant tumor. We report the case of a 61-year-old male who presented with postprandial abdominal pain, recurrent vomiting, transfusion-dependent anemia, and significant weight loss. Contrast-enhanced CT showed a large circumferential mass in the proximal jejunum, mesenteric thrombosis, and a hepatic lesion suggestive of metastasis. Anterograde double-balloon enteroscopy revealed an ulceroinfiltrative lesion approximately 40 cm distal to the ligament of Treitz, with friable mucosa and prominent vasculature. Targeted biopsies confirmed a high-grade pleomorphic LMS. The patient underwent segmental enterectomy and partial colectomy; histopathology confirmed high-grade LMS with hepatic metastasis. He recovered uneventfully and was referred for oncologic care. This case highlights the importance of early endoscopic evaluation in patients with obscure anemia and subacute obstructive symptoms. Deep enteroscopy enabled histologic confirmation and timely surgical intervention in a lesion beyond the reach of conventional endoscopy.

## Introduction

Primary leiomyosarcoma (LMS) of the small intestine is an exceptionally rare mesenchymal tumor, accounting for less than 2% of gastrointestinal malignancies and approximately 0.1-3% of small bowel tumors [1, 2]. The ileum is the most frequently affected segment, followed by the jejunum [2]. Due to its nonspecific presentation, such as abdominal pain, anemia, weight loss, gastrointestinal bleeding, or subacute obstruction, diagnosis is often delayed, and many cases are identified only at a locally advanced or metastatic stage [3].

Unlike gastrointestinal stromal tumors (GISTs), which benefit from molecular characterization and targeted therapies, LMS lacks KIT and DOG1 expression and typically follows a more aggressive course with limited systemic treatment options [4, 5]. Diagnosis relies on histopathologic identification of spindle cells with smooth muscle differentiation, confirmed by desmin and smooth muscle actin positivity, and negative staining for CD117, DOG1, and S100 [4, 5].

While cross-sectional imaging helps localize lesions and assess spread, it rarely offers a definitive diagnosis [4, 6]. Device-assisted enteroscopy plays a key role by enabling direct visualization and tissue sampling of small bowel lesions beyond the reach of conventional endoscopy, especially in patients with obscure bleeding, anemia, or suspected neoplasia [7].

## Case presentation

A 61-year-old man with a history of hypertension, type 2 diabetes mellitus, and chronic hepatitis C (diagnosed in 2002) presented in December 2024 with a three-month history of progressive postprandial abdominal pain, recurrent vomiting, unintentional 12 kg weight loss, fatigue, and intermittent fever. He also described episodic symptoms of partial bowel obstruction, including colicky abdominal pain, distension, and postprandial emesis. His surgical history included inguinal and umbilical herniorrhaphies performed in May 2024, with no cross-sectional imaging available at that time. Laboratory tests revealed transfusion-dependent anemia, consistent with iron deficiency in the setting of chronic disease.

**Figure 1:**
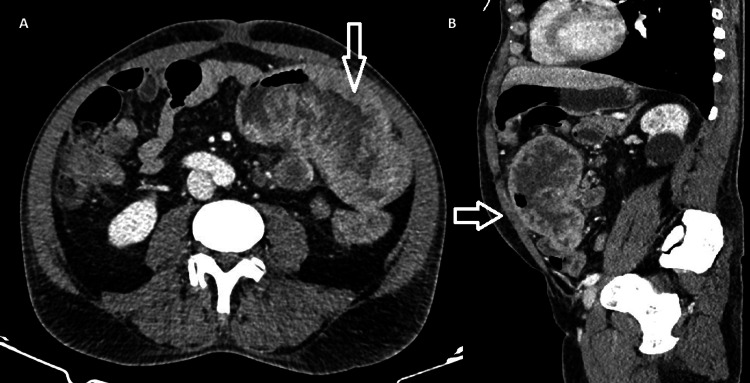
Contrast-enhanced computed tomography (CT) of the abdomen demonstrating a jejunal leiomyosarcoma. (A) Axial view showing a large, heterogeneous, and ulceroinfiltrative mass (arrow) arising from the proximal jejunum, exhibiting irregular wall thickening and central areas of necrosis. Adjacent mesenteric fat stranding and necrotic lymphadenopathy are also noted. (B) Sagittal reconstruction revealing the exophytic and transmural extension of the lesion (arrow), causing partial luminal narrowing without complete obstruction.

Contrast-enhanced abdominal and pelvic CT (Figure 1) in February 2025 revealed a 15 cm circumferential mass in the proximal jejunum, approximately 15 cm distal to the ligament of Treitz. Associated findings included mild upstream bowel stasis, thrombosis of jejunal branches of the superior mesenteric vein, and necrotic mesenteric lymphadenopathy (up to 3.3 cm). A 4.2 cm hypovascular lesion in hepatic segment VI was also identified, raising suspicion for metastasis.

**Figure 2:**
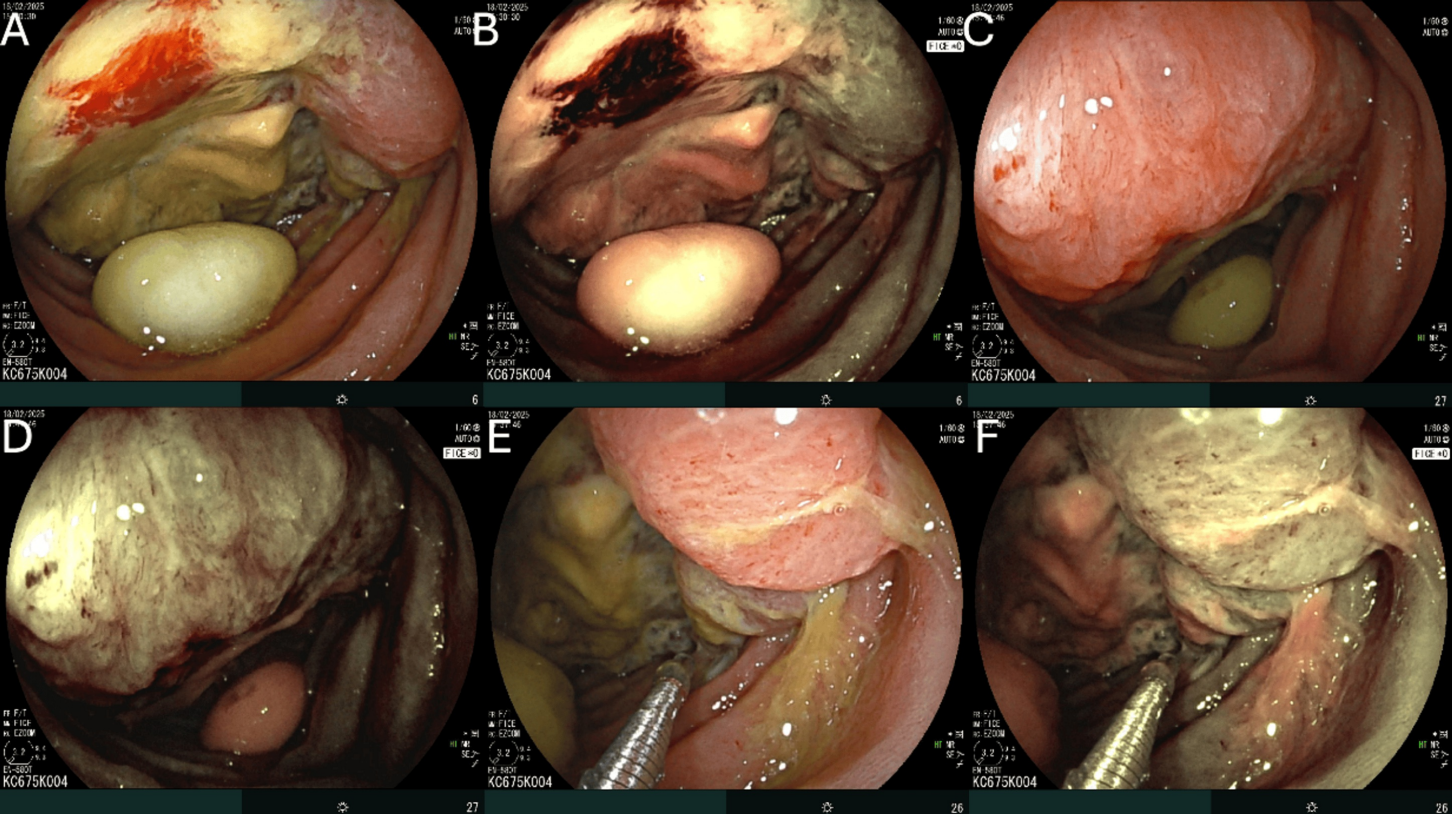
Endoscopic findings during anterograde double-balloon enteroscopy using white-light imaging and FICE. (A, B) Ulceroinfiltrative jejunal mass approximately 40 cm distal to the ligament of Treitz, causing partial luminal obstruction and showing friable mucosa with fibrin-covered ulcerations, under white-light and FICE imaging, respectively. (C, D) Same lesion demonstrating irregular borders, prominent and tortuous vasculature, and areas of mucosal necrosis under both imaging modalities. (E, F) Forceps biopsy being performed on the ulcerated portion of the lesion, shown with white-light and FICE enhancement, respectively. FICE, flexible spectral imaging color enhancement.

Subsequent anterograde double-balloon enteroscopy (DBE) using a Fujifilm EN-580T enteroscope (Fujifilm Corporation, Tokyo, Japan) allowed direct visualization of the lesion (Figure 2), which appeared as an extensive ulceroinfiltrative mass approximately 40 cm distal to the ligament of Treitz. The lesion was characterized by irregular and friable mucosa, prominent tortuous vessels, and a fibrin-covered ulcerated center. Due to the presence of dense adhesions, endoscope progression beyond the lesion was not possible. Multiple biopsies were obtained from the affected area.

**Figure 3:**
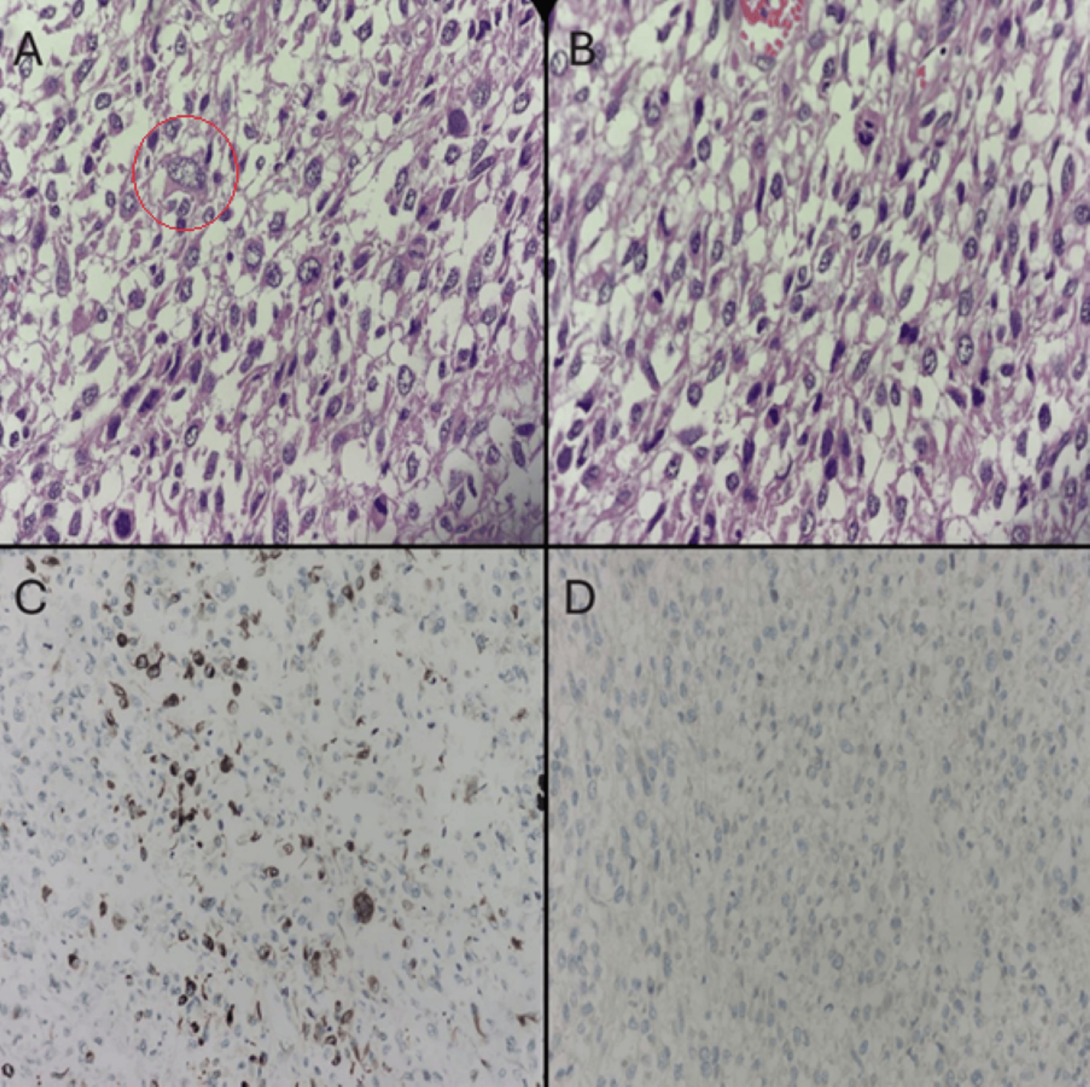
Histopathological and immunohistochemical features of jejunal leiomyosarcoma. (A) Hematoxylin–eosin staining showing spindle cell proliferation arranged in fascicles (×200; red circle). (B) High-power view demonstrating marked nuclear pleomorphism and frequent mitotic figures (×400). (C) Immunohistochemical staining for desmin showing focal cytoplasmic positivity, supporting smooth muscle differentiation (×200). (D) Negative immunoreactivity for DOG1, helping exclude gastrointestinal stromal tumor (GIST) (×200).

Histopathological analysis (Figure 3) revealed a poorly differentiated spindle cell neoplasm arranged in short fascicles, with marked nuclear pleomorphism and a high mitotic rate (>20 mitoses per 10 high-power fields). Immunohistochemistry showed focal cytoplasmic positivity for desmin, confirming smooth muscle differentiation, while staining for CD117, DOG1, CD34, cytokeratin, and S100 was negative, excluding epithelial, neural, and gastrointestinal stromal tumors. Ki-67 immunostaining was not performed, however, the high mitotic index and extensive necrosis were consistent with a high-grade leiomyosarcoma.

Given the diagnostic findings and the symptomatic obstruction, the patient underwent exploratory laparotomy in early March. Intraoperatively, a large exophytic jejunal tumor was identified, infiltrating both the duodenal wall and the transverse colon, along with segment VI liver involvement consistent with metastatic spread. The surgical team performed a segmental enterectomy and partial colectomy with a hand-sewn duodenojejunal anastomosis and terminal colostomy.

Histopathological examination of the resected specimen revealed a 15 cm pleomorphic high-grade sarcoma infiltrating the full thickness of the jejunal wall and extending to the subserosa of the transverse colon, with approximately 35% tumor necrosis and 21 mitoses per 5 high-power fields (equivalent to approximately 42 mitoses per 10 mm², field diameter 0.55 mm). Immunohistochemical staining demonstrated focal desmin positivity, while markers for epithelial, melanocytic, GIST-related (CD117, DOG1, CD34), and skeletal muscle differentiation were negative. These findings confirmed the diagnosis of high-grade jejunal leiomyosarcoma. All surgical margins were free of tumor cells, and no lymphovascular or perineural invasion was observed. Nineteen regional lymph nodes were negative for metastasis.

The postoperative course was marked by gradual clinical improvement, with recovery of oral intake and stable colostomy function. He was discharged in good general condition with planned outpatient follow-up in oncology, surgery, and cardiology services for further management of metastatic jejunal leiomyosarcoma.

## Discussion

Primary leiomyosarcoma (LMS) of the small intestine is a rare malignancy that often presents at an advanced stage due to its deep anatomical location and vague clinical features, such as abdominal pain, anemia, and subocclusive symptoms [1-3]. Hematogenous dissemination commonly leads to liver or lung metastases. Although contrast-enhanced computed tomography (CT) remains the first-line imaging modality, it often cannot differentiate LMS from other spindle cell tumors like GISTs or sarcomas [4].

In this case, cross-sectional imaging identified a proximal jejunal mass with associated thrombosis and suspected liver metastasis, but the definitive diagnosis was only possible through anterograde double-balloon enteroscopy (DBE). Enteroscopy revealed a friable ulceroinfiltrative lesion, allowing for targeted biopsies that confirmed high-grade LMS. Compared to capsule endoscopy, DBE offers both visualization and histologic sampling, which is vital for deep small bowel lesions inaccessible to conventional endoscopes [7, 8]. In selected centers, DBE shows a diagnostic yield above 85% for small bowel tumors [7].

Histological diagnosis relies on the identification of pleomorphic spindle cells with mitotic activity and necrosis, alongside immunohistochemical profiling. LMS typically expresses smooth muscle markers (desmin, SMA) and lacks expression of CD117, DOG1, CD34, and S100, helping distinguish it from GISTs, sarcomatoid carcinomas, and melanomas [4, 5]. In this patient, the tumor exhibited a high mitotic index (>20/10 HPF), 35% necrosis, and focal desmin positivity, consistent with a high-grade neoplasm.

Prognosis in small bowel LMS depends on factors such as tumor size, grade, mitotic count, and presence of metastases. Data from the Surveillance, Epidemiology, and End Results (SEER) database confirm worse outcomes in patients with high-grade tumors or distant spread [5, 6]. Surgical resection with negative margins is the cornerstone of treatment. In non-metastatic disease, complete excision provides the best chance of long-term survival [6]. The role of adjuvant chemotherapy remains uncertain, as prospective data are lacking. However, doxorubicin-based regimens, including combinations with ifosfamide, are commonly used. Alternatives such as gemcitabine and docetaxel may be appropriate for patients ineligible for anthracyclines [8, 9].

While small bowel leiomyosarcoma is most often diagnosed after surgical resection, endoscopic confirmation before surgery remains exceptional. Robles et al. reviewed malignant small bowel tumors diagnosed by double-balloon enteroscopy and found that leiomyosarcomas accounted for less than 2% of all cases, with most diagnoses established postoperatively [10]. These data underscore how uncommon preoperative histologic confirmation of small bowel LMS can be. Therefore, the present case is notable for demonstrating successful endoscopic diagnosis through double-balloon enteroscopy, enabling prompt surgical management and contributing to the limited literature documenting this approach in leiomyosarcoma of the small intestine.

This case underscores the indispensable role of device-assisted enteroscopy in diagnosing rare small bowel tumors. Early histological confirmation guided prompt surgical intervention, highlighting the importance of integrating enteroscopy into the diagnostic workup of patients with obscure gastrointestinal bleeding, unexplained anemia, or suspected small bowel neoplasms [9, 10].

## Conclusions

Primary jejunal leiomyosarcoma is a rare and often late diagnosed malignancy due to its nonspecific presentation and inaccessible location. This case underscores the clinical utility of device-assisted enteroscopy, which enabled early visualization and histological confirmation of a deeply located small bowel tumor. By providing access for targeted biopsy beyond the reach of standard endoscopy, enteroscopy plays a central role in the diagnostic pathway of small bowel neoplasms. Prompt recognition and tissue diagnosis are critical, as timely surgical intervention and multidisciplinary coordination remain essential in managing such aggressive tumors.
